# Supplementary material for: Study protocol for a randomised, controlled platform trial estimating the effect of autobiographical Memory Flexibility training (MemFlex) on relapse of recurrent major depressive disorder
Source: BMJ Open. 2018 Jan 29;8(1):e018194. doi: 10.1136/bmjopen-2017-018194 (PMC5829844; doi:10.1136/bmjopen-2017-018194)
Supplement: Author's manuscript [file bmjopen-2017-018194.draft_revisions.pdf]

BMJ Open is committed to open peer review. As part of this commitment we make the peer review history of every article we publish publicly available.

When an article is published we post the peer reviewers' comments and the authors' responses online. We also post the versions of the paper that were used during peer review. These are the versions that the peer review comments apply to.

The versions of the paper that follow are the versions that were submitted during the peer review process. They are not the versions of record or the final published versions. They should not be cited or distributed as the published version of this manuscript.

BMJ Open is an open access journal and the full, final, typeset and author-corrected version of record of the manuscript is available on our site with no access controls, subscription charges or pay-per-view fees (http://bmjopen.bmj.com).

If you have any questions on BMJ Open's open peer review process please email editorial.bmjopen@bmj.com

BMJ Open

Study protocol for a randomised, controlled platform trial estimating the effect of autobiographical Memory Flexibility Training (MemFlex) on relapse of recurrent Major Depressive Disorder

Journal:	*BMJ Open*
Manuscript ID	bmjopen-2017-018194
Article Type:	Protocol
Date Submitted by the Author:	12-Jun-2017
Complete List of Authors:	Hitchcock, Caitlin; University of Cambridge, MRC Cognition and Brain Sciences Unit; MRC Cognition and Brain Sciences Uni<br>Gormley, Siobhan; University of Cambridge, MRC Cognition and Brain Sciences Unit<br>O Leary, Cliodhna; University of Cambridge, MRC Cognition and Brain Sciences Unit<br>Rodrigues, Evangeline; University of Cambridge, MRC Cognition and Brain Sciences Unit<br>Chadwick, Isobel; University of Cambridge, MRC Cognition and Brain Sciences Unit<br>Griffiths, Kirsty; University of Cambridge, MRC Cognition and Brain Sciences Unit<br>Gillard, Julia; University of Cambridge, MRC Cognition and Brain Sciences Unit<br>Watson, Peter; MRC Cognition and Brain Sciences Unit<br>Hammond, Emily; University of Cambridge, MRC Cognition and Brain Sciences Unit<br>Werner-Seidler, Aliza ; University of New South Wales, The Black Dog Institute<br>Dalgleish, Tim; Medical Research Council Cognition and Brain Sciences Unit,
**Primary Subject Heading**:	Mental health
Secondary Subject Heading:	Mental health
Keywords:	Depression & mood disorders < PSYCHIATRY, Clinical trials < THERAPEUTICS, Adult psychiatry < PSYCHIATRY

SCHOLARONE™ Manuscripts

Study protocol for a randomised, controlled platform trial estimating the effect of autobiographical Memory Flexibility Training (MemFlex) on relapse of recurrent Major Depressive Disorder

Caitlin Hitchcock [1]

Siobhan Gormley [1]

Cliodhna O'Leary[1]

Evangeline Rodrigues[1]

Isobel Chadwick [1]

Kirsty Griffiths [1]

Julia Gillard [1]

Peter Watson [1]

Emily Hammond [1,3]

Aliza Werner-Seidler [2]

Tim Dalgleish [1, 4]

[1] Medical Research Council Cognition and Brain Sciences Unit, University of Cambridge

[2] The Black Dog Institute, Sydney

[3] University of Exeter

[4] Cambridgeshire and Peterborough NHS Foundation Trust

Correspondence

Caitlin Hitchcock Caitlin.hitchcock@mrc-cbu.cam.ac.uk

or Tim Dalgleish tim.dalgleish@mrc-cbu.cam.ac.uk

For peer review only

Introduction: Major depressive disorder (MDD) is a chronic condition. Although current treatment approaches are effective in reducing acute depressive symptoms, rates of relapse are high. Chronic and inflexible retrieval of autobiographical memories, and in particular a bias toward negative and overgeneral memories, is a reliable predictor of relapse. This randomised controlled single blind trial will determine whether a therapist-guided self-help intervention to ameliorate autobiographical memory biases using Memory Flexibility Training (MemFlex) will increase the experience of depression free days, relative to a Psychoeducation control condition, in the twelve months following intervention. **Methods and Analysis**: Individuals (aged 18 and above) with a diagnosis of recurrent MDD will be recruited when remitted from a major depressive episode. Participants will be randomly allocated to complete four weeks of a workbook providing either MemFlex training, or Psychoeducation on factors that increase risk of relapse. Assessment of diagnostic status, self-report depressive symptoms, depression free days and cognitive risk factors for depression will be completed post-intervention, and at six and twelve months follow-up. The cognitive target of MemFlex will be change in memory flexibility on the Autobiographical Memory Test- Alternating Instructions. The primary clinical endpoints will be the number of depression free days in the twelve months following workbook completion, and time to depressive relapse. **Ethics and Dissemination**: Ethics approval has been granted by the NHS National Research Ethics Committee (East of England, 11/H0305/1). Results from this study will provide a point-estimate of the effect of MemFlex on depressive relapse, which will be used to inform a fully-powered trial evaluating the potential of MemFlex as an effective, low-cost, and low-intensity option for reducing relapse of MDD. **Trial registration**: This trial is pre-registered at clinicaltrials.gov, identifier NCT02614326.

For peer review only

Strengths and limitations

- This is an early-stage platform trial seeking to establish an estimate of effect size to power a later-stage trial
- The intervention condition will be compared to an active control condition

For peer review only

Depression is a recurrent and pervasive condition which dramatically reduces quality of life for the individual and costs the health system in the United Kingdom an estimated £7.5 billion per year (McCrone, Dhanasiri, Patel, Knapp, & Lawton-Smith, 2008). Although psychological approaches to treating depression are effective in reducing current symptoms (Butler, Chapman, Forman, & Beck, 2006), relapse rates are high. On average, those with major depressive disorder (MDD) will experience ten separate depressive episodes across their lifespan (Judd, 1997), and symptom severity increases with each experienced episode (Kessing, 2008). As such, reducing depressive relapse is a key challenge for health systems. Broadly, depressive risk is increased by the chronic use of maladaptive cognitive styles which promote depressogenic thoughts, attributions, and beliefs (Burcusa & Iacono, 2007), thus intervention to reduce the chronicity of these cognitive styles may thereby break the vicious cycle of depression. Importantly, as cognitive risk factors for depression remain evident when depressive symptoms remit, it may be possible to complete interventions to shift these factors when the individual is psychologically well, and more able to engage in therapy. Further exploration of low-cost and low-intensity options for reducing these cognitive risk factors may have important implications for limiting depressive relapse and subsequent costs for the individual and health care system. This trial will investigate the efficacy of a low-intensity programme, Memory Flexibility Training (MemFlex), which aims to reduce one such risk factor- inflexible retrieval of autobiographical memories.

Autobiographical memory, that is, memory for personal life experiences, is integral in forming one's sense of self (Conway & Pleydell-Pearce, 2000), and disruptions in autobiographical memory increase risk of depression (for review see Dalgleish & Werner-Seidler, 2014). Autobiographical information is stored in a fluid manner, and can therefore be retrieved in a variety of formats, including as specific, single incident events that are rich in detail (e.g., *when I forgot my purse and couldn't buy my train ticket*), or as generalisations

For peer review only - http://bmjopen.bmj.com/site/about/guidelines.xhtml

that summarise consistency across a category of events (e.g., *catching the train to work every day*). Impaired flexibility in moving between these memory types, and in shifting the emotional valence of the information retrieved, appears to be a key predictor of the first onset of depression (Hipwell, Sapotichne, Klostermann, Battista, & Keenan, 2011) and also of future depressive episodes in those with a history of recurrent depression (for a meta-analysis see Sumner, Griffith, & Mineka, 2010). That is, this reduced flexibility is not simply a by-product of the depressed state, but rather predicts the course of depression.

Reduced flexibility is expressed through the chronic over-retrieval of autobiographical memories that are: a) negative in emotional valence; and b) overgeneral in nature. Regarding valence, depressive risk is increased by a habitual and inflexible inclination towards negative memories, and subsequently, a neglect for positively- and neutrally-valenced memories (Gotlib & Joormann, 2010). However, it is not simply that negative information is retrieved more readily, but that when positive information is recalled, it is less vivid (Werner-Seidler & Moulds, 2011) and therefore has an attenuated impact on affect, reducing the emotional benefits that are gained from recalling positive memories (Joormann & Siemer, 2004; Werner-Seidler, Tan & Dalgleish, 2016). In this way, biased memory retrieval can impair the ability to repair transient low mood (Teasdale & Dent, 1987). In addition, the absence of vivid, positive memories of oneself and a continual emphasis on negative autobiographical information reinforces the negative self-schemata that maintain and promote depression (Beck, 1967).

Inflexible movement between specific and general memory types also increases risk for depression. A tendency toward general memory recollection and reduced retrieval of specific memories is evident in those with current depression, however, the overgeneral retrieval style does not remit with depressive symptoms (Williams et al., 2007), suggesting that reduced specific recall is a durable cognitive marker for depression that may not be

For peer review only

shifted by current psychological treatments. Repeated recall of overgeneral summaries may promote depression by reinforcing overly general (negative) attributions regarding the self (e.g., I always fail) and the world (e.g., the world is an unfair place). Previous research has also demonstrated that impaired retrieval of specific memories impacts a number of intermediate factors that promote depression. Abstract and overgeneral memory recall intensifies rumination (Watkins et al., 2012), which in turn creates negative affect, impairs mood regulation, and is itself a risk factor for recurrent depression (Nolen-Hoeksema, 1991). Impaired retrieval of specific memories also reduces the ability to establish intimacy in social communication (Beike, Brandon, & Cole, 2016), impairs problem solving (Jing, Madore, & Schacter, 2016), and limits the ability to appropriately restrain the overly general, negative self-judgements that are characteristic of depression (Hitchcock, Rees, & Dalgleish, 2017).

As reduced flexibility in the use of negative and overgeneral memory retrieval styles both independently predicts depression symptoms (Dritschel, Beltsos, & McClintock, 2014; Sumner et al., 2010) and may also interact with other risk factors (e.g., stress; Sumner et al., 2011) to trigger a depressive episode, intervening to reduce bias and improve memory flexibility may thereby reduce depressive risk. There is some evidence to suggest a positive impact of targeting these factors individually, such as by reducing overgeneral memory through memory specificity training (e.g., Neshat-Doost et al., 2013; Raes et al., 2009; see Hitchcock, Werner-Seidler, Blackwell, & Dalgleish, 2017). However, simultaneously targeting these factors may reduce compounded effects, and promote flexibility in the use of other depressogenic cognitive styles (Nolen-Hoeksema, Wisco, & Lyubomirsky, 2008). To this end, we have previously piloted a novel intervention which aims to improve cognitive flexibility in those experiencing recurrent depression (Hitchcock et al., 2016). Memory Flexibility training (MemFlex) uses autobiographical memory as a modality to train negative and overgeneral processing styles. The workbook-based programme is presented in eight self-

For peer review only

guided sessions completed over a one month period, and involves completion of memory retrieval exercises that aim to improve concrete, specific processing, thereby reducing overgeneral, abstract processing, and improve access to neutral and positive emotional material, thereby reducing negative bias. In an initial uncontrolled trial with those in remission from MDD, MemFlex was effective in reducing overgeneral memory, rumination, and cognitive avoidance, and also improved social problem solving from pre- to post-intervention (Hitchcock et al., 2016). An ongoing randomised controlled trial is comparing the effect of MemFlex to an active psychological intervention for those experiencing a current Major Depressive Episode (see Hitchcock et al., 2015 for trial protocol), however results from the uncontrolled trial suggest that MemFlex may prove beneficial not only for current symptoms of depression, but for also reducing cognitive risk factors for depressive relapse.

Preliminary evidence of an effect on multiple depressive risk factors provides a solid foundation for a larger trial of MemFlex determining the efficacy of the programme in relapse prevention, against an active control group (Medical Research Council, 2000). In line with recommendations for the phase-based development and evaluation of novel interventions, we will complete an early-stage, randomised controlled platform trial with the primary aim of estimating the likely effect size of MemFlex on measures of depressive relapse, which can then be used to inform a later-stage, fully-powered trial. This study will therefore estimate the effect of MemFlex on the recurrence of depression over a twelve-month period, relative to an active control condition – Psychoeducation. We hypothesise that individuals completing MemFlex when they are currently remitted from depression will experience a greater number of depression free days in the twelve months following workbook completion and demonstrate a longer period of time until depressive relapse, compared to those who complete Psychoeducation.

For peer review only

Methods and Analysis

Study Design

The design is a single-blind, participant-level randomised controlled superiority trial comparing MemFlex to Psychoeducation. All participants will provide informed consent to complete a four-week programme, consisting of an initial face-to-face session, followed by eight self-guided workbook sessions presenting either the Psychoeducation or MemFlex materials. Participants will be assessed at baseline, post-intervention, at six months follow-up and at twelve months follow-up.

Participants and recruitment

Inclusion criteria are a minimum age of 18 years and a primary diagnosis of Major Depressive Disorder (MDD), as assessed using the Structured Clinical Interview for DSM Disorders (SCID; First et al., 2015). Upon study entry, participants must not be currently experiencing a Major Depressive Episode. That is, participants must be in partial or full remission from MDD, as indexed by the Longitudinal Interval Follow-up Evaluation (LIFE; Keller et al., 1987) for the SCID. Additional exclusion criteria are experience of another current mood disorder, psychosis, personality disorder, or current drug or alcohol dependence. Those experiencing significant cognitive impairment, as assessed through self-report, will also be excluded. Participants may continue with any current recovery-maintenance medication or psychological interventions (e.g., booster sessions, mindfulness practice) while participating in the study.

We will recruit through three avenues in Cambridge, UK. First, participants will be invited to contact us through advertisements in the general community (e.g., posters in GP surgeries) and the local newspaper. All those who contact us will be sent further information about the study, and invited to complete an initial telephone screening. We will also recruit from our database of participants with depression who are interested in taking part in

For peer review only

research. All participants on this database have previously completed the SCID and meet criteria for MDD. These participants will be telephoned or emailed to make them aware of the study, and all those who express an interest in participating will complete the LIFE to determine current depression and wider diagnostic status. Finally, we will recruit patients who have expressed an interest in participating in research from local mental health services. Clinical care teams will be made aware of the study, and will be encouraged to ask their patients if they would like to take part in research. If the patient expresses an interest in research, their clinical care team will pass on our information sheet.

Participant allocation

Random allocation to condition using computer-generated, quasi-random numbers will completed by the trial statistician (PW), who is blind to study objectives. Once a participant has provided informed consent and completed the pre-intervention assessment, the researcher completing the initial session will open a sealed, opaque envelope to reveal condition allocation. This process is demonstrated in the CONSORT diagram (see Figure 1).

Interventions

MemFlex. We have previously piloted the MemFlex programme with individuals in remission from depression (Hitchcock et al., 2016). The programme aims to improve the flexibility with which individuals move between specific and general personal memories, and to train away a bias toward negative memories. This is achieved through repeated completion of cued recall tasks which use positive and neutral cues. The training exercises are introduced in an initial face-to-face session with a researcher, and the participant is then required to complete twice-weekly sessions of training over a four-week period. During the initial session, the researcher provides information about the role of autobiographical memory in depression, including the different types of autobiographical memories (e.g., specific event memories, and the generalised themes that link together a series of events) and their

For peer review only

presumed role and importance in daily life. The rationale for MemFlex is then introduced. The researcher then assists the individual to complete practice exercises, and provides feedback and encouragement to ensure that the individual understands the tasks. The session lasts between 45 and 60 minutes. The participant will also receive a telephone call from the researcher halfway through the time set to complete the workbook to answer any questions, and address any difficulties the individual may be having with the workbook. The participant is also encouraged to contact the researcher with any questions about the workbook content.

The MemFlex workbook is separated into eight sessions. The early sessions of the workbook focus on reducing the bias toward negative and general memories experienced in depression by promoting the retrieval of both specific and general memories of neutral and positive valence. This involves repeated cued-recall exercises using neutral and positive cues. Next, the workbook encourages the individual to elaborate the specific emotional details attached to positive memories, again using repeated retrieval tasks. This seeks to improve the reduced vividness of positive memories experienced in depression (Werner-Seidler & Moulds, 2011). The final workbook sessions involve completion of exercises that require the participant to explicitly link specific event memories with a general theme, and in reverse, to identify individual event memories that form a general theme. Exercises are repeated throughout the workbook to give participants practice in using the different skills, with the aim of promoting flexible movement between memory types.

Psychoeducation. Participants in the Psychoeducation condition will also attend a 45-60 minute face-to-face session, followed by the completion of a workbook over a four week period. The initial session will provide information on the causes of depressive relapse, and the individual's experience of relapse will be discussed between the researcher and participant. The eight-session workbook will then be introduced.

For peer review only

The Psychoeducation workbook is a relapse-focussed adaptation of that used in a previous trial for those with MDD (Hitchcock et al., 2015). The workbook presents theory on different cognitive and lifestyle factors that are associated with depression which may persist between depressive episodes, and promote the experience of low mood in the future. These include poor sleeping patterns, worry, and perfectionism. After the relevant theory is explained, each component of the workbook will offer tips for improving each factor, and an exercise which encourages the participant to reflect on whether these factors occur in their life. Four multiple-choice questions are also asked at the end of each session to ensure participant engagement, and to match the completion of workbook exercises against the MemFlex condition.

Treatment integrity. Researchers will be trained and supervised by a clinical psychologist (CH) to administer the manualised initial sessions. Sessions will be audio-recorded, and 15% of these will rated for adherence to the manual by a researcher independent from the project. Progress on the workbook will be checked halfway through the four-weeks provided for workbook completion. To ensure adherence, the workbook will also be viewed at post-intervention to ensure all materials have been attempted. The number of completed sessions will be recorded.

Measures

Cognitive target. We anticipate that the MemFlex programme will improve, and maintain improvement in, memory flexibility relative to Psychoeducation. We will therefore administer the Autobiographical Memory Test with Alternating Instructions (AMT-AI; Dritschel, Beltsos, & McClintock, 2014) at all assessments. The AMT-AI requires individuals to recall either specific or general memories in response to neutral, positive, and negative cue words. Participants are asked to retrieve specific memories to a block of six cue words, general memories for a block of six cues, and finally a block of twelve cues in which

For peer review only

the individual must alternate between retrieval of specific and general memories. The cues are randomised to blocks, and the order in which the blocks are presented is also randomised. The main outcomes are the number and proportion of correct responses for each block (i.e., specific, general, alternating).

Primary clinical outcomes. The primary clinical outcomes are number of depression free days from post-intervention to twelve month follow-up, and number of days until depressive relapse, both according to the LIFE.

Secondary clinical outcomes. The secondary clinical outcomes are depressive status at twelve month follow-up, again measured using the LIFE, and symptoms of depression at twelve month follow-up as indexed on the Beck Depression Inventory II (BDI-II; Beck, Steer, & Brown, 1996).

Additional process measures. We have previous evidence to suggest that MemFlex will impact rumination, social problem solving, and cognitive avoidance in samples remitted from depression (Hitchcock et al., 2016). We will therefore assess whether these factors may mediate the impact of MemFlex on our primary and secondary outcomes. Rumination will be measured using the Rumination Response Scale (Treynor, Gonzalez, & Nolen-Hoeksema, 2003), cognitive avoidance will be measured using the Cognitive Avoidance Questionnaire (Sexton & Dugas, 2008), and social problem solving will be indexed on the short version of the Means-Ends Problem Solving task (Lyubomirsky & Nolen-Hoeksema, 1995). We will also assess verbal fluency (using the Verbal Fluency Task; Tombaugh, Kozak, & Rees, 1999) to determine whether the programme impacts the fluent retrieval of verbal information. Each of these measures possesses adequate psychometric properties and has been previously used in evaluation of autobiographical memory interventions (e.g., Dalgleish & Werner-Seidler, 2014; Hitchcock et al., 2015, Hitchcock et al., 2016). Parallel forms of these measures will be counterbalanced and administered at all time points.

For peer review only

Methodological Aspects

Power analysis. As this is an exploratory, early stage randomised controlled platform trial (in accordance with recommendations for the evaluation of complex interventions; MRC, 2000), we aim to generate estimates of the range of likely effect sizes of MemFlex (relative to Psychoeducation) on our primary clinical outcomes. This can then be used to inform a power calculation for a fully-powered later-phase trial. Our prior experience with autobiographical memory based interventions for depression (Hitchcock et al., 2015, Hitchcock et al., 2016, Neshat-Doost et al., 2013, Travers-Hill et al., 2017) suggests that 25 participants per condition will provide a plausible range of point estimates. Given the long-term follow-up we implement, we aim to recruit 35 participants in each condition (70 participants in total) to allow for up to 25% attrition.

Data collection and blinding. Participants will be assessed at baseline, and then again at post-intervention, and at six and twelve month follow-ups after the intervention has been completed. All sessions will be completed at the MRC Cognition and Brain Sciences Unit in Cambridge, United Kingdom, except in the case that a participant moves away from Cambridge before the completion of all assessments. In these cases, six- and twelve-month follow-up assessments may be completed over the telephone. As the AMT-AI is unable to be completed over the telephone, it will not be administered at follow-up for these participants. Research staff completing the assessments will be supervised by a clinical psychologist (CH) and inter-rater reliability will be completed for the AMT-AI and MEPS to ensure data quality and protection from bias. Bias will also be protected against at all assessments through use of research staff whom are blind to experimental condition.

Statistical analysis plan. The trial statistician is blind to study objectives, and will complete all analyses in accordance with CONSORT standards. No interim analyses are planned during data collection. Between-group difference on our primary clinical outcome of

For peer review only

number of depression free days will be analysed using an independent samples t test, covarying for any baseline differences. Time to depressive relapse will be compared between groups using Cox regression survival analysis. Analysis of our cognitive target variable, secondary clinical outcomes and additional process measures will be analysed using analysis of variance (ANOVA) with time (pre -, post-, six and twelve follow-up) as a within-subjects factor, and condition (MemFlex, Psychoeducation) as a between subjects factor. If present, any baseline differences will again be used as co-variates. Diagnostic status at twelve month follow-up will analysed using logistic regression. Residual depression symptoms and number of previous episodes (as an index of depression severity), and score on the AMT-AI (as an index of bias severity) at pre-intervention will be explored as moderators of treatment efficacy. Finally, we will explore mechanisms of change by determining whether intervention effects are mediated by change on the AMT-AI and the other process measures. Intent-to-treat analysis will be used for those lost to attrition by using multiple imputation to account for missing data.

Trial management. The trial management committee will be formed by TD, CH, and trial statistician (PW). The trial coordinator (CH) will complete data checks to ensure data quality.

Ethics and Dissemination

This trial has been approved by the NHS National Research Ethics Committee (East of England, 11/H0305/1). All individuals will provide informed consent prior to participation, and Medical Research Council, National Health Service and professional ethical guidelines will be adhered to throughout the study. Any necessary amendments to this protocol will be published in the trial manuscript. No restrictions have been placed on the publication of results. In addition to publication of the trial paper, results will be shared in written form to trial participants, and through seminars to clinicians and the general public.

For peer review only

Safety aspects. In the unlikely occurrence of adverse events, the Medical Research Council's structured risk assessment protocols will be used to manage risk. All risk issues will be managed by the trial co-ordinator (CH), in consultation with TD, both of whom are clinical psychologists experienced in treatment of depression. Any adverse events will be reported to the research ethics committee, and will be reported in the trial manuscript.

Confidentiality and access to data. Only the trial co-ordinator and trial statistician will have access to the final dataset. Data will be securely stored in de-identified form onsite, and in encrypted electronic format. Personally identifiable information will be stored separately to the trial data, and will only be accessible via a dedicated access server. Once the data have been analysed and the trial is published, the final dataset will be deposited in de-identified form into the research unit's online data repository, along with the utilised statistical code.

Funding. This work is supported by a grant to TD from the UK Medical Research Council, grant number MC_US_A060_0019.

Trial registration. This trial was pre-registered in January 2016 at clinicaltrials.gov, identifier NCT02614326.

Discussion

Major depression is predicted to be the largest global burden of disease by 2030 (Mathers & Loncar, 2006). Reducing the recurrence of depression will therefore be a key issue for the coming years. This study will estimate the effect of a novel intervention, MemFlex, which aims to reduce negative and overgeneral memory biases, and thereby risk of depressive relapse. In simultaneously targeting these factors, the programme may reduce both direct and compounded effects on depressive relapse. This trial aims to estimate the range of likely effects of MemFlex, to provide a platform for a later stage, fully powered trial, and to begin to explore mechanisms of change. If effective, MemFlex may hold promise as a low-

For peer review only

cost and low-intensity option for reducing depressive relapse as the self-guided, workbook format of the programme would be simple and cost-effective to provide to individuals upon discharge from a treatment service, or potentially as an adjunct to complex interventions such as cognitive behavioural therapy. As the pressure that depression places on health care systems is expected to increase (World Health Organisation, 2004), further development of low-cost relapse prevention programmes will be vital in improving access to psychological services and quality of life for those experiencing chronic and recurrent depression.

Authors' contributions: CH co-developed the study design, co-wrote the intervention materials, and co-wrote the manuscript. SG, CO, ER, IC, KG, and JG are contributing to data collection for the trial. PW is the trial statistician. EH and AWS co-wrote the intervention materials. TD co-developed the study design, co-wrote intervention materials and co-wrote the manuscript. The authors have no conflict of interest to declare.

For peer review only

References

Beck, A. T. (1967). *Depression: Clinical, Experimental, and Theoretical Aspects*: Hoeber Medical Division, Harper & Row.

Beck, A. T., Steer, R., & Brown, G. (1996). *Manual for the Beck Depression Inventory-II*. San Antonio, TX:: Psychological Corporation.

Beike, D. R., Brandon, N. R., & Cole, H. E. (2016). Is sharing specific autobiographical memories a distinct form of self-disclosure? *Journal of Experimental Psychology: General*, *145*. 434-450. doi:10.1037/xge0000143

Burcusa, S. L., & Iacono, W. G. (2007). Risk for recurrence in depression. *Clinical Psychology Review, 27*, 959-985. doi:10.1016/j.cpr.2007.02.005

Butler, A. C., Chapman, J. E., Forman, E. M., & Beck, A. T. (2006). The empirical status of cognitive-behavioral therapy: A review of meta-analyses. *Clinical Psychology Review, 26*, 17-31. doi:10.1016/j.cpr.2005.07.003

Conway, M. A., & Pleydell-Pearce, C. W. (2000). The construction of autobiographical memories in the self-memory system. *Psychological Review, 107*, 261-288. doi:10.1037/0033-295X.107.2.261

Dalgleish, T., & Werner-Seidler, A. (2014). Disruptions in autobiographical memory processing in depression and the emergence of memory therapeutics. *Trends in Cognitive Sciences, 18*, 596-604. doi:10.1016/j.tics.2014.06.010

Dritschel, B., Beltsos, S., & McClintock, S. M. (2014). An “alternating instructions” version of the Autobiographical Memory Test for assessing autobiographical memory specificity in non-clinical populations. *Memory, 22*, 881-889. doi:10.1080/09658211.2013.839710

Gotlib, I., & Joormann, J. (2010). Cognition and depression: Current status and future directions. *Annual Review of Clinical Psychology, 6*, 285-312.

For peer review only

Hipwell, A. E., Sapotichne, B., Klostermann, S., Battista, D., & Keenan, K. (2011). Autobiographical memory as a predictor of depression vulnerability in girls. *Journal of Clinical Child & Adolescent Psychology, 40*, 254-265. doi:10.1080/15374416.2011.546037

Hitchcock, C., Hammond, E., Rees, C., Panesar, I., Watson, P., Werner-Seidler, A., & Dalgleish, T. (2015). Memory Flexibility training (MemFlex) to reduce depressive symptomatology in individuals with major depressive disorder: Study protocol for a randomised controlled trial. *Trials, 16*, 1-8. doi:10.1186/s13063-015-1029-y

Hitchcock, C., Mueller, V., Hammond, E., Rees, C., Werner-Seidler, A., & Dalgleish, T. (2016). The effects of autobiographical memory flexibility (MemFlex) training: An uncontrolled trial in individuals in remission from depression. *Journal of Behavior Therapy and Experimental Psychiatry, 52*, 92-98. doi:10.1016/j.jbtep.2016.03.012

Hitchcock, C., Rees, C., & Dalgleish, T. (2017). The devil's in the detail: Accessibility of specific personal memories supports rose-tinted self-generalizations in mental health and toxic self-generalizations in clinical depression. *Journal of Experimental Psychology: General.*

Jing, H. G., Madore, K. P., & Schacter, D. L. (2016). Worrying about the future: An episodic specificity induction impacts problem solving, reappraisal, and well-being. *Journal of Experimental Psychology: General*, *145*, 402-418. doi:10.1037/xge0000142

Joormann, J., & Siemer, M. (2004). Memory accessibility, mood regulation, and dysphoria: difficulties in repairing sad mood with happy memories? *Journal of Abnormal Psychology, 113*, 179. doi:10.1037/0021-843X.113.2.179

Judd, L. L. (1997). The clinical course of unipolar major depressive disorders. *Archives of General Psychiatry, 54*, 989-991. doi:10.1001/archpsyc.1997.01830230015002.

For peer review only

Keller, M. B., Lavori, P. W., Friedman, B., Nielsen, E., Endicott, J., McDonald-Scott, P., & Andreasen, N. C. (1987). The Longitudinal Interval Follow-up Evaluation. A comprehensive method for assessing outcome in prospective longitudinal studies. *Archives of General Psychiatry, 44*, 540-548. doi:10.1001/archpsyc.1987.01800180050009

Kessing, L. V. (2008). Severity of depressive episodes during the course of depressive disorder. *The British Journal of Psychiatry, 192*, 290-293. doi:10.1192/bjp.bp.107.038935

Lyubomirsky, S., & Nolen-Hoeksema, S. (1995). Effects of self-focused rumination on negative thinking and interpersonal problem solving. *Journal of Personality and Social Psychology, 69*, 176-190. doi:10.1037/0022-3514.69.1.176

McCrone, P. R., Dhanasiri, S., Patel, A., Knapp, M., & Lawton-Smith, S. (2008). *Paying the price: The cost of mental health care in England to 2026*. King's Fund. Retrieved from www.kingsfund.org.uk/sites/files/kf/Paying-the-Price-the-cost-of-mental-health-care-England-2026-McCrone-Dhanasiri-Patel-Knapp-Lawton-Smith-Kings-Fund-May-2008_0.pdf

Mathers, C.D., & Loncar, D. (2006). Projections of global mortality and burden of disease from 2002 to 2030. *PLoS Medicine, 3.* e442. doi: 10.1371/journal.pmed.0030442

Sexton, K. A., & Dugas, M. J. (2008). The cognitive avoidance questionnaire: validation of the English translation. *Journal of Anxiety Disorders, 22*, 355-370. doi:10.1016/j.janxdis.2007.04.005

Sumner, J. A., Griffith, J. W., & Mineka, S. (2010). Overgeneral autobiographical memory as a predictor of the course of depression: A meta-analysis. *Behaviour Research and Therapy, 48*, 614-625. doi:10.1016/j.brat.2010.03.013

For peer review only

Sumner, J. A., Griffith, J. W., Mineka, S., Rekart, K. N., Zinbarg, R. E., & Craske, M. G. (2011). Overgeneral autobiographical memory and chronic interpersonal stress as predictors of the course of depression in adolescents. *Cognition and Emotion, 25*, 183-192. doi:10.1080/02699931003741566

Tombaugh, T. N., Kozak, J., & Rees, L. (1999). Normative data stratified by age and education for two measures of verbal fluency: FAS and animal naming. *Archives of Clinical Neuropsychology, 14*, 167-177. doi:10.1016/S0887-6177(97)00095-4

Travers-Hill, E., Dunn, B. D., Hoppitt, L., Hitchcock, C., & Dalgleish, T. (2017). Beneficial effects of training in self-distancing and perspective broadening for people with a history of recurrent depression. *Behaviour Research and Therapy, 95*, 19-28. doi: 10.1016/j.brat.2017.05.008

Treynor, W., Gonzalez, R., & Nolen-Hoeksema, S. (2003). Rumination reconsidered: A psychometric analysis. *Cognitive Therapy and Research, 27*, 247-259. doi:10.1023/A:1023910315561

Werner-Seidler, A., & Moulds, M. L. (2011). Autobiographical memory characteristics in depression vulnerability: Formerly depressed individuals recall less vivid positive memories. *Cognition & Emotion, 25*, 1087-1103. doi:10.1080/02699931.2010.531007

Werner-Siedler, A., Tan, L., & Dalgleish T. (2016). The vicissitudes of positive autobiographical recollection as an emotion regulation strategy in depression. *Clinical Psychological Science,* doi: 2167702616647922.

Williams, J. M. G., Barnhofer, T., Crane, C., Herman, D., Raes, F., Watkins, E. R., & Dalgleish, T. (2007). Autobiographical memory specificity and emotional disorder. *Psychological Bulletin, 133*, 122-148. doi:10.1037/0033-2909.133.1.122

For peer review only

World Health Organisation (2004). *The Global Burden of Disease.* Retrieved from http://www.who.int/healthinfo/global_burden_disease/GBD_report_2004update_full.pdf

For peer review only

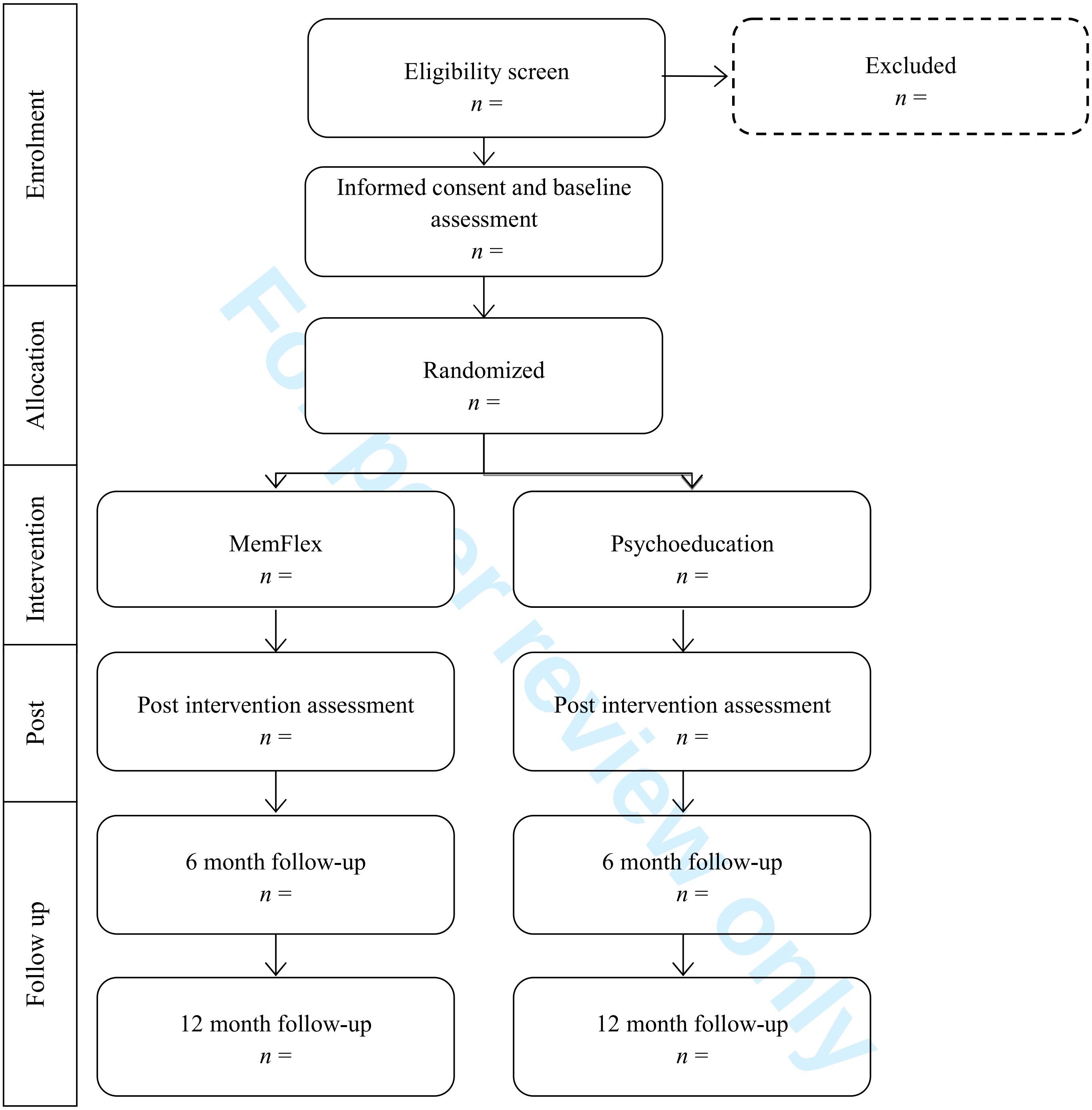

Figure 1. Flow chart of study design (CONSORT diagram). All outcome and process measures will be completed at all time points.

For peer review only

STANDARD PROTOCOL ITEMS: RECOMMENDATIONS FOR INTERVENTIONAL TRIALS

SPIRIT 2013 Checklist: Recommended items to address in a clinical trial protocol and related documents*

Section/item	Item No	Description	Addressed on page number
**Administrative information**			
Title	1	Descriptive title identifying the study design, population, interventions, and, if applicable, trial acronym	______1_______
Trial registration	2a	Trial identifier and registry name. If not yet registered, name of intended registry	______2______
	2b	All items from the World Health Organization Trial Registration Data Set	_2 (refer to online record)_______
Protocol version	3	Date and version identifier	______1_______
Funding	4	Sources and types of financial, material, and other support	_____15______
Roles and responsibilities	5a	Names, affiliations, and roles of protocol contributors	___1, 16______
	5b	Name and contact information for the trial sponsor	____1,15_______
	5c	Role of study sponsor and funders, if any, in study design; collection, management, analysis, and interpretation of data; writing of the report; and the decision to submit the report for publication, including whether they will have ultimate authority over any of these activities	____16_______
	5d	Composition, roles, and responsibilities of the coordinating centre, steering committee, endpoint adjudication committee, data management team, and other individuals or groups overseeing the trial, if applicable (see Item 21a for data monitoring committee)	_____14______

For peer review only - http://bmjopen.bmj.com/site/about/guidelines.xhtml

For peer review only

**Introduction**			
Background and rationale	6a	Description of research question and justification for undertaking the trial, including summary of relevant studies (published and unpublished) examining benefits and harms for each intervention	_____4-7___
	6b	Explanation for choice of comparators	____7______
Objectives	7	Specific objectives or hypotheses	____7________
Trial design	8	Description of trial design including type of trial (eg, parallel group, crossover, factorial, single group), allocation ratio, and framework (eg, superiority, equivalence, noninferiority, exploratory)	___8_______
**Methods: Participants, interventions, and outcomes**			
Study setting	9	Description of study settings (eg, community clinic, academic hospital) and list of countries where data will be collected. Reference to where list of study sites can be obtained	___8________
Eligibility criteria	10	Inclusion and exclusion criteria for participants. If applicable, eligibility criteria for study centres and individuals who will perform the interventions (eg, surgeons, psychotherapists)	____8_______
Interventions	11a	Interventions for each group with sufficient detail to allow replication, including how and when they will be administered	__9-11_______
	11b	Criteria for discontinuing or modifying allocated interventions for a given trial participant (eg, drug dose change in response to harms, participant request, or improving/worsening disease)	_____NA______
	11c	Strategies to improve adherence to intervention protocols, and any procedures for monitoring adherence (eg, drug tablet return, laboratory tests)	_____10______
	11d	Relevant concomitant care and interventions that are permitted or prohibited during the trial	______8______
Outcomes	12	Primary, secondary, and other outcomes, including the specific measurement variable (eg, systolic blood pressure), analysis metric (eg, change from baseline, final value, time to event), method of aggregation (eg, median, proportion), and time point for each outcome. Explanation of the clinical relevance of chosen efficacy and harm outcomes is strongly recommended	_____11-12____
Participant timeline	13	Time schedule of enrolment, interventions (including any run-ins and washouts), assessments, and visits for participants. A schematic diagram is highly recommended (see Figure)	_____Figure 1__

For peer review only - http://bmjopen.bmj.com/site/about/guidelines.xhtml

Sample size	14	Estimated number of participants needed to achieve study objectives and how it was determined, including clinical and statistical assumptions supporting any sample size calculations	_____13_____
Recruitment	15	Strategies for achieving adequate participant enrolment to reach target sample size	____8_______

Methods: Assignment of interventions (for controlled trials)

Allocation:

Sequence generation	16a	Method of generating the allocation sequence (eg, computer-generated random numbers), and list of any factors for stratification. To reduce predictability of a random sequence, details of any planned restriction (eg, blocking) should be provided in a separate document that is unavailable to those who enrol participants or assign interventions	______9______
Allocation concealment mechanism	16b	Mechanism of implementing the allocation sequence (eg, central telephone; sequentially numbered, opaque, sealed envelopes), describing any steps to conceal the sequence until interventions are assigned	_____9____
Implementation	16c	Who will generate the allocation sequence, who will enrol participants, and who will assign participants to interventions	___9______
Blinding (masking)	17a	Who will be blinded after assignment to interventions (eg, trial participants, care providers, outcome assessors, data analysts), and how	___9, 13________
	17b	If blinded, circumstances under which unblinding is permissible, and procedure for revealing a participant's allocated intervention during the trial	_____NA_______

Methods: Data collection, management, and analysis

Data collection methods	18a	Plans for assessment and collection of outcome, baseline, and other trial data, including any related processes to promote data quality (eg, duplicate measurements, training of assessors) and a description of study instruments (eg, questionnaires, laboratory tests) along with their reliability and validity, if known. Reference to where data collection forms can be found, if not in the protocol	__11-12___
	18b	Plans to promote participant retention and complete follow-up, including list of any outcome data to be collected for participants who discontinue or deviate from intervention protocols	____11-12____

For peer review only - http://bmjopen.bmj.com/site/about/guidelines.xhtml

Data management	19	Plans for data entry, coding, security, and storage, including any related processes to promote data quality (eg, double data entry; range checks for data values). Reference to where details of data management procedures can be found, if not in the protocol	___13____
Statistical methods	20a	Statistical methods for analysing primary and secondary outcomes. Reference to where other details of the statistical analysis plan can be found, if not in the protocol	___13-14_______
	20b	Methods for any additional analyses (eg, subgroup and adjusted analyses)	__13-14________
	20c	Definition of analysis population relating to protocol non-adherence (eg, as randomised analysis), and any statistical methods to handle missing data (eg, multiple imputation)	_____14______
**Methods: Monitoring**			
Data monitoring	21a	Composition of data monitoring committee (DMC); summary of its role and reporting structure; statement of whether it is independent from the sponsor and competing interests; and reference to where further details about its charter can be found, if not in the protocol. Alternatively, an explanation of why a DMC is not needed	__14_______
	21b	Description of any interim analyses and stopping guidelines, including who will have access to these interim results and make the final decision to terminate the trial	_____14______
Harms	22	Plans for collecting, assessing, reporting, and managing solicited and spontaneously reported adverse events and other unintended effects of trial interventions or trial conduct	_____15_____
Auditing	23	Frequency and procedures for auditing trial conduct, if any, and whether the process will be independent from investigators and the sponsor	______11_____
**Ethics and dissemination**			
Research ethics approval	24	Plans for seeking research ethics committee/institutional review board (REC/IRB) approval	_____14______
Protocol amendments	25	Plans for communicating important protocol modifications (eg, changes to eligibility criteria, outcomes, analyses) to relevant parties (eg, investigators, REC/IRBs, trial participants, trial registries, journals, regulators)	______14______

For peer review only - http://bmjopen.bmj.com/site/about/guidelines.xhtml

Consent or assent	26a	Who will obtain informed consent or assent from potential trial participants or authorised surrogates, and how (see Item 32)	_____9______
	26b	Additional consent provisions for collection and use of participant data and biological specimens in ancillary studies, if applicable	____NA______
Confidentiality	27	How personal information about potential and enrolled participants will be collected, shared, and maintained in order to protect confidentiality before, during, and after the trial	____15______
Declaration of interests	28	Financial and other competing interests for principal investigators for the overall trial and each study site	____16_______
Access to data	29	Statement of who will have access to the final trial dataset, and disclosure of contractual agreements that limit such access for investigators	_____15_____
Ancillary and post-trial care	30	Provisions, if any, for ancillary and post-trial care, and for compensation to those who suffer harm from trial participation	____15______
Dissemination policy	31a	Plans for investigators and sponsor to communicate trial results to participants, healthcare professionals, the public, and other relevant groups (eg, via publication, reporting in results databases, or other data sharing arrangements), including any publication restrictions	____15______
	31b	Authorship eligibility guidelines and any intended use of professional writers	_______16_____
	31c	Plans, if any, for granting public access to the full protocol, participant-level dataset, and statistical code	_____NA______
**Appendices**			
Informed consent materials	32	Model consent form and other related documentation given to participants and authorised surrogates	_Available on request_
Biological specimens	33	Plans for collection, laboratory evaluation, and storage of biological specimens for genetic or molecular analysis in the current trial and for future use in ancillary studies, if applicable	_____NA______

*It is strongly recommended that this checklist be read in conjunction with the SPIRIT 2013 Explanation & Elaboration for important clarification on the items. Amendments to the protocol should be tracked and dated. The SPIRIT checklist is copyrighted by the SPIRIT Group under the Creative Commons "Attribution-NonCommercial-NoDerivs 3.0 Unported" license.

For peer review only - http://bmjopen.bmj.com/site/about/guidelines.xhtml

BMJ Open

Study protocol for a randomised, controlled platform trial estimating the effect of autobiographical Memory Flexibility Training (MemFlex) on relapse of recurrent Major Depressive Disorder

Journal:	*BMJ Open*
Manuscript ID	bmjopen-2017-018194.R1
Article Type:	Protocol
Date Submitted by the Author:	25-Oct-2017
Complete List of Authors:	Hitchcock, Caitlin; University of Cambridge, MRC Cognition and Brain Sciences Unit; MRC Cognition and Brain Sciences Uni<br>Gormley, Siobhan; University of Cambridge, MRC Cognition and Brain Sciences Unit<br>O Leary, Cliodhna; University of Cambridge, MRC Cognition and Brain Sciences Unit<br>Rodrigues, Evangeline; University of Cambridge, MRC Cognition and Brain Sciences Unit<br>Chadwick, Isobel; University of Cambridge, MRC Cognition and Brain Sciences Unit<br>Griffiths, Kirsty; University of Cambridge, MRC Cognition and Brain Sciences Unit<br>Gillard, Julia; University of Cambridge, MRC Cognition and Brain Sciences Unit<br>Watson, Peter; MRC Cognition and Brain Sciences Unit<br>Hammond, Emily; University of Cambridge, MRC Cognition and Brain Sciences Unit<br>Werner-Seidler, Aliza ; University of New South Wales, The Black Dog Institute<br>Dalgleish, Tim; Medical Research Council Cognition and Brain Sciences Unit,
**Primary Subject Heading**:	Mental health
Secondary Subject Heading:	Mental health
Keywords:	Depression & mood disorders < PSYCHIATRY, Clinical trials < THERAPEUTICS, Adult psychiatry < PSYCHIATRY

SCHOLARONE™
Manuscripts

Study protocol for a randomised, controlled platform trial estimating the effect of autobiographical Memory Flexibility Training (MemFlex) on relapse of recurrent Major Depressive Disorder

Caitlin Hitchcock [1]

Siobhan Gormley [1]

Cliodhna O'Leary[1]

Evangeline Rodrigues[1]

Isobel Chadwick [1]

Kirsty Griffiths [1]

Julia Gillard [1]

Peter Watson [1]

Emily Hammond [1,3]

Aliza Werner-Seidler [2]

Tim Dalgleish [1, 4]

[1] Medical Research Council Cognition and Brain Sciences Unit, University of Cambridge

[2] The Black Dog Institute, Sydney

[3] University of Exeter

[4] Cambridgeshire and Peterborough NHS Foundation Trust

Correspondence

Caitlin Hitchcock Caitlin.hitchcock@mrc-cbu.cam.ac.uk

or Tim Dalgleish tim.dalgleish@mrc-cbu.cam.ac.uk

For peer review only

Introduction: Major depressive disorder (MDD) is a chronic condition. Although current treatment approaches are effective in reducing acute depressive symptoms, rates of relapse are high. Chronic and inflexible retrieval of autobiographical memories, and in particular a bias toward negative and overgeneral memories, is a reliable predictor of relapse. This randomised controlled single blind trial will determine whether a therapist-guided self-help intervention to ameliorate autobiographical memory biases using Memory Flexibility Training (MemFlex) will increase the experience of depression free days, relative to a Psychoeducation control condition, in the twelve months following intervention. **Methods and Analysis**: Individuals (aged 18 and above) with a diagnosis of recurrent MDD will be recruited when remitted from a major depressive episode. Participants will be randomly allocated to complete four weeks of a workbook providing either MemFlex training, or Psychoeducation on factors that increase risk of relapse. Assessment of diagnostic status, self-report depressive symptoms, depression free days and cognitive risk factors for depression will be completed post-intervention, and at six and twelve months follow-up. The cognitive target of MemFlex will be change in memory flexibility on the Autobiographical Memory Test- Alternating Instructions. The primary clinical endpoints will be the number of depression free days in the twelve months following workbook completion, and time to depressive relapse. **Ethics and Dissemination**: Ethics approval has been granted by the NHS National Research Ethics Committee (East of England, 11/H0305/1). Results from this study will provide a point-estimate of the effect of MemFlex on depressive relapse, which will be used to inform a fully-powered trial evaluating the potential of MemFlex as an effective, low-cost, and low-intensity option for reducing relapse of MDD. **Trial registration**: This trial is pre-registered at clinicaltrials.gov, identifier NCT02614326.

For peer review only

Strengths and limitations

- This early-stage platform trial seeks to establish an estimate of effect size to power a later-stage trial
- The intervention condition will be compared to an active control condition
- The hardcopy workbook format limits the ability to track completion of the intervention sessions in real-time
- The absence of booster sessions following workbook completion may limit the long-term efficacy of the intervention

For peer review only

Depression is a recurrent and pervasive condition which dramatically reduces quality of life for the individual and costs the health system in the United Kingdom an estimated £7.5 billion per year (1). Although psychological approaches to treating depression are effective in reducing current symptoms (2), relapse rates are high. On average, those with major depressive disorder (MDD) will experience ten separate depressive episodes across their lifespan (3), and symptom severity increases with each experienced episode (4). As such, reducing depressive relapse is a key challenge for health systems. Broadly, depressive risk is increased by the chronic use of maladaptive cognitive styles which promote depressogenic thoughts, attributions, and beliefs (5), thus intervention to reduce the chronicity of these cognitive styles may thereby break the vicious cycle of depression. Importantly, as cognitive risk factors for depression remain evident when depressive symptoms remit, it may be possible to complete interventions to shift these factors when the individual is psychologically well, and more able to engage in therapy. Further exploration of low-cost and low-intensity options for reducing these cognitive risk factors may have important implications for limiting depressive relapse and subsequent costs for the individual and health care system. This trial will investigate the efficacy of a low-intensity programme, Memory Flexibility Training (MemFlex), which aims to reduce one such risk factor- inflexible retrieval of autobiographical memories.

Autobiographical memory, that is, memory for personal life experiences, is integral in forming one's sense of self (6), and disruptions in autobiographical memory increase risk of depression (for review see 7). Autobiographical information is stored in a fluid manner, and can therefore be retrieved in a variety of formats, including as specific, single incident events that are rich in detail (e.g., *when I forgot my purse and couldn't buy my train ticket*), or as generalisations that summarise consistency across a category of events (e.g., *catching the train to work every day*). Memory flexibility refers to the ability to successfully navigate the

For peer review only

autobiographical information database to deliberately retrieve memories varying in detail and emotional valence. In depression, flexible movement within the autobiographical information database is compromised (8, 9), resulting in relative difficulties in retrieving both general and specific memories (8-11). This can create functional impairment as both types of memory are necessary in daily cognitive skills; for example, specific memories can help to guide problem solving (12) while general memories encode regularities of experiences that can be used to inform judgements of the self, world, and others (13, 14). Impaired recall of specific memories is a key predictor of the first onset of depression (15) and also of future depressive episodes in those with a history of recurrent depression (for meta-analysis see 16), although comparable studies examining the predictive effects of impaired generality or flexibility have yet to be carried out.

A core feature of reduced flexibility is the chronic over-retrieval of autobiographical memories that are: a) negative in emotional valence; and b) overgeneral in nature. Regarding valence, depressive risk is increased by a habitual and inflexible inclination towards negative memories, and subsequently, a neglect for positively- and neutrally-valenced memories (17). However, it is not simply that negative information is retrieved more readily, but that when positive information is recalled, it is less vivid (18) and therefore has an attenuated impact on affect, reducing the emotional benefits that are gained from recalling positive memories (19). In this way, biased memory retrieval can impair the ability to repair transient low mood (20). In addition, the absence of vivid, positive memories of oneself and a continual emphasis on negative autobiographical information reinforces the negative self-schemata that maintain and promote depression (21).

Difficulties in intentional autobiographical retrieval also increases risk for depression. A tendency toward general memory recollection and reduced retrieval of specific memories is evident in those with current depression, however, the overgeneral retrieval style does not

For peer review only

remit with depressive symptoms (11), suggesting that reduced specific recall is a durable cognitive marker for depression (16) that may not be shifted by current psychological treatments (22), though this has not yet been well explored. Repeated recall of overgeneral summaries may promote depression by reinforcing overly general (negative) attributions regarding the self (e.g., I always fail) and the world (e.g., the world is an unfair place). Previous research has also demonstrated that impaired retrieval of specific memories impacts a number of intermediate factors that promote depression. Abstract and overgeneral memory recall intensifies rumination (23), which in turn creates negative affect, impairs mood regulation, and is itself a risk factor for recurrent depression (24). Impaired retrieval of specific memories also reduces the ability to establish intimacy in social communication (25), impairs problem solving (26), and limits the ability to appropriately restrain the overly general, negative self-judgements that are characteristic of depression (27).

As reduced flexibility in the use of negative and overgeneral memory retrieval styles both independently promote depression symptoms (16, 28) and may also interact with other risk factors (e.g., stress; 29) to trigger a depressive episode, intervening to reduce bias and improve memory flexibility may thereby reduce depressive risk. There is some evidence to suggest a positive impact of targeting these factors individually, such as by reducing overgeneral memory through memory specificity training (e.g., (30, 31); see (32)). However, simultaneously targeting these factors may reduce compounded effects, and promote flexibility in the use of other depressogenic cognitive styles (33). In addition, explicitly training improved recall of positive, general memories (e.g., of kindness, determination, caring relationships) may help to support re-initiation of the overly general, positive self-judgements that are associated with mental health (for review see 34).

To this end, we have previously piloted a novel intervention which aims to improve cognitive flexibility in those experiencing recurrent depression (35). Memory Flexibility

For peer review only

training (MemFlex) uses autobiographical memory as a modality to train negative and overgeneral processing styles. The workbook-based programme is presented in eight self-guided sessions completed over a one month period, and involves completion of memory retrieval exercises that aim to improve concrete, specific processing, thereby reducing overgeneral, abstract processing, and improve access to neutral and positive emotional material, thereby reducing negative bias. In an initial uncontrolled trial with those in remission from MDD, MemFlex was effective in reducing overgeneral memory (d= 0.48), rumination (d= 0.29), and cognitive avoidance (d= 0.18), and also improved social problem solving (d= 0.55) from pre- to post-intervention (35). An ongoing randomised controlled trial is comparing the effect of MemFlex to an active psychological intervention for those experiencing a current Major Depressive Episode (see (36) for trial protocol), however results from the uncontrolled trial suggest that MemFlex may prove beneficial not only for current symptoms of depression, but for also reducing cognitive risk factors for depressive relapse.

Preliminary evidence of an effect on multiple depressive risk factors provides a solid foundation for a larger trial of MemFlex determining the efficacy of the programme in relapse prevention, against an active control group (37). In line with recommendations for the phase-based development and evaluation of novel interventions, we will complete an early-stage, randomised controlled platform trial with the primary aim of estimating the likely effect size of MemFlex on measures of depressive relapse, which can then be used to inform a later-stage, fully-powered trial. This study will therefore estimate the effect of MemFlex on the recurrence of depression over a twelve-month period, relative to an active control condition – Psychoeducation. We hypothesise effect sizes in favour of MemFlex, such that individuals completing MemFlex when they are currently remitted from depression will experience a greater number of depression free days in the twelve months following

For peer review only

workbook completion and demonstrate a longer period of time until depressive relapse, compared to those who complete Psychoeducation.

Methods and Analysis

Study Design

The design is a single-blind, participant-level randomised controlled superiority trial comparing MemFlex to Psychoeducation. All participants will provide informed consent to complete a four-week programme, consisting of an initial face-to-face session, followed by eight self-guided workbook sessions presenting either the Psychoeducation or MemFlex materials. Participants will be assessed at baseline, post-intervention, at six months follow-up and at twelve months follow-up.

Participants and recruitment

Inclusion criteria are a minimum age of 18 years and a primary diagnosis of Major Depressive Disorder (MDD), as assessed using the Structured Clinical Interview for DSM Disorders (SCID; (38)). Upon study entry, participants must not be currently experiencing a Major Depressive Episode. That is, participants must be in partial or full remission from MDD, as indexed by the Longitudinal Interval Follow-up Evaluation (LIFE; (39)) for the SCID. Additional exclusion criteria are prior completion of any autobiographical memory-based training programme, experience of another current mood disorder, psychosis, personality disorder, or current drug or alcohol dependence. Those experiencing significant cognitive impairment, as assessed through self-report (via an open ended question asking whether the individual has ever experienced an intellectual disability or traumatic brain injury), will also be excluded. Participants may continue with any current recovery-maintenance medication or psychological interventions (e.g., booster sessions, mindfulness practice) while participating in the study.

For peer review only

We will recruit through three avenues in Cambridge, UK. First, participants will be invited to contact us through advertisements in the general community (e.g., posters in GP surgeries) and the local newspaper. All those who contact us will be sent further information about the study, and invited to complete an initial telephone screening. We will also recruit from our database of participants with depression who are interested in taking part in research. All participants on this database have previously completed the SCID and meet criteria for MDD. These participants will be telephoned or emailed to make them aware of the study, and all those who express an interest in participating will complete the LIFE to determine current depression and wider diagnostic status. Finally, we will recruit patients who have expressed an interest in participating in research from local mental health services. Clinical care teams will be made aware of the study, and will be encouraged to ask their patients if they would like to take part in research. If the patient expresses an interest in research, their clinical care team will pass on our information sheet.

Participant allocation

Random allocation to condition using computer-generated, quasi-random numbers will completed by the trial statistician (PW), who is blind to study objectives. Once a participant has provided informed consent and completed the pre-intervention assessment, the researcher completing the initial session will open a sealed, opaque envelope to reveal condition allocation. This process is demonstrated in the CONSORT diagram (see Figure 1).

Interventions

MemFlex. We have previously piloted the MemFlex programme with individuals in remission from depression (35). The intervention will be delivered as per the pilot trial and the trial evaluating the intervention in those with current depression (36).The programme aims to improve the flexibility of autobiographical retrieval processes, to improve the ease with which individuals can access and move between specific and general personal

For peer review only

memories, and to train away a bias toward negative memories. This is achieved through repeated completion of cued recall tasks which use positive and neutral cues. The training exercises are introduced in an initial face-to-face session with a researcher, and the participant is then required to complete twice-weekly sessions of training over a four-week period. During the initial session, the researcher provides information about the role of autobiographical memory in depression, including the different types of autobiographical memories (e.g., specific event memories, and the generalised themes that link together a series of events) and their presumed role and importance in daily life. The rationale for MemFlex is then introduced. The researcher then assists the individual to complete practice exercises, and provides feedback and encouragement to ensure that the individual understands the tasks. The session lasts between 45 and 60 minutes. The participant will also receive a telephone call from the researcher halfway through the time set to complete the workbook to answer any questions, and address any difficulties the individual may be having with the workbook. The participant is also encouraged to contact the researcher with any questions about the workbook content.

The MemFlex workbook is separated into eight sessions. The early sessions of the workbook focus on reducing the bias toward negative and general memories experienced in depression by promoting the retrieval of both specific and general memories of neutral and positive valence. This involves repeated cued-recall exercises using neutral and positive cues. Next, the workbook encourages the individual to elaborate the specific emotional details attached to positive memories, again using repeated retrieval tasks. This seeks to improve the reduced vividness of positive memories experienced in depression (18). The final workbook sessions involve completion of exercises that require the participant to explicitly link specific event memories with a general theme, and in reverse, to identify individual event memories that form a general theme. To do this, individuals must explicitly switch between retrieving

For peer review only

specific and general memories to the same cue, thereby promoting flexibility in movement between the different memory types. When completed with positive cues, this type of exercise also aims to promote positive generalisations about the self. Exercises are repeated throughout the workbook to give participants practice in using the different skills. There are additional exercises at the end of the workbook that the participant can choose to do after the study has ended. While participants are encouraged to continue to apply their new skills in their everyday life, completion of the additional workbook exercises is optional.

Psychoeducation. Participants in the Psychoeducation condition will also attend a 45-60 minute face-to-face session, followed by the completion of a workbook over a four week period. The initial session will provide information on the causes of depressive relapse, and the individual's experience of relapse will be discussed between the researcher and participant. The eight-session workbook will then be introduced.

The Psychoeducation workbook is a relapse-focussed adaptation of that used in a previous trial for those with MDD (36). The workbook presents theory on different cognitive and lifestyle factors that are associated with depression which may persist between depressive episodes, and promote the experience of low mood in the future. These include poor sleeping patterns, worry, and perfectionism. After the relevant theory is explained, each component of the workbook will offer tips for improving each factor, and an exercise which encourages the participant to reflect on whether these factors occur in their life. Four multiple-choice questions are also asked at the end of each session to ensure participant engagement, and to match the completion of workbook exercises against the MemFlex condition. Interventions are matched for time burden.

Treatment integrity. Both workbooks are available from the corresponding authors upon request, and will be made available online if their efficacy is demonstrated. Researchers will be trained and supervised by a clinical psychologist (CH) to administer the manualised

For peer review only

initial sessions. Sessions will be audio-recorded, and 15% of these will rated for adherence to the manual by a researcher independent from the project. At the end of the initial session, participants will complete the Credibility Expectancy Questionnaire – Patient Version (40) to index any difference in treatment expectations between the two intervention conditions. Progress on the workbook will be checked halfway through the four-weeks provided for workbook completion. If the individual is behind on workbook completion, any difficulties in completing the workbook will be briefly addressed, and trial management records will be updated with an anticipated finish date. A member of the research team will call the individual the week before the anticipated finish date to set an appointment time for the post-intervention assessment. To ensure adherence, the workbook will also be viewed at post-intervention to ensure all materials have been attempted. The number of completed sessions will be recorded.

Measures

Cognitive target. We anticipate that the MemFlex programme will improve, and maintain improvement in, memory flexibility relative to Psychoeducation. We will therefore administer the Autobiographical Memory Test with Alternating Instructions (AMT-AI; (8) at all assessments. The AMT-AI requires individuals to recall either specific or general memories in response to neutral, positive, and negative cue words. Participants are asked to retrieve specific memories to a block of six cue words, general memories for a block of six cues, and finally a block of twelve cues in which the individual must alternate between retrieval of specific and general memories. The cues are randomised to blocks, and the order in which the blocks are presented is also randomised. The main outcomes are the number and proportion of correct responses for each block (i.e., specific, general, alternating).

For peer review only

Primary clinical outcomes. The primary clinical outcomes are number of depression free days from post-intervention to twelve month follow-up, and number of days until depressive relapse, both according to the LIFE.

Secondary clinical outcomes. The secondary clinical outcomes are depressive status at twelve month follow-up, again measured using the LIFE, and symptoms of depression at twelve month follow-up as indexed on the Beck Depression Inventory II (BDI-II; (41)).

Additional process measures. We have previous evidence to suggest that MemFlex will impact rumination, social problem solving, and cognitive avoidance in samples remitted from depression (35). We will therefore assess whether these factors may mediate the impact of MemFlex on our primary and secondary outcomes. Rumination will be measured using the Rumination Response Scale (42), cognitive avoidance will be measured using the Cognitive Avoidance Questionnaire (43), and social problem solving will be indexed on the short version of the Means-Ends Problem Solving task (44). We will also assess verbal fluency (using the Verbal Fluency Task; 45) to determine whether the programme impacts the fluent retrieval of verbal information. Each of these measures possesses adequate psychometric properties and has been previously used in evaluation of autobiographical memory interventions (e.g., (7); (35, 36)). Parallel forms of these measures will be counterbalanced and administered at all time points.

Methodological Aspects

Power analysis. As this is an exploratory, early stage randomised controlled platform trial (in accordance with recommendations for the evaluation of complex interventions; MRC, 2000), we aim to generate estimates of the range of likely effect sizes of MemFlex (relative to Psychoeducation) on our primary clinical outcomes. This can then be used to inform a power calculation for a fully-powered later-phase trial. Our prior experience with autobiographical memory based interventions for depression (e.g., 30, 35, 36, 46) suggests

For peer review only

that 25 participants per condition will provide a plausible range of point estimates. Given the long-term follow-up we implement, we aim to recruit 35 participants in each condition (70 participants in total) to allow for up to 25% attrition.

Data collection and blinding. Participants will be assessed at baseline, and then again at post-intervention, and at six and twelve month follow-ups after the intervention has been completed. All sessions will be completed at the MRC Cognition and Brain Sciences Unit in Cambridge, United Kingdom, except in the case that a participant moves away from Cambridge before the completion of all assessments. In these cases, six- and twelve-month follow-up assessments may be completed over the telephone. As the AMT-AI is unable to be completed over the telephone, it will not be administered at follow-up for these participants. Research staff completing the assessments will be supervised by a clinical psychologist (CH) and inter-rater reliability will be completed for the AMT-AI and MEPS to ensure data quality and protection from bias. Bias will also be protected against at all assessments through use of research staff whom are blind to experimental condition.

Statistical analysis plan. The trial statistician is blind to study objectives, and will complete all analyses in accordance with CONSORT standards. No interim analyses are planned during data collection. Between-group difference on our primary clinical outcome of number of depression free days will be analysed using an independent samples t test, or analysis of co-variance if necessary to account for any baseline differences. Time to depressive relapse will be compared between groups using Cox regression survival analysis. Analysis of our cognitive target variable, secondary clinical outcomes and additional process measures will be analysed using analysis of variance (ANOVA) with time (pre -, post-, six and twelve follow-up) as a within-subjects factor, and condition (MemFlex, Psychoeducation) as a between subjects factor. Any baseline differences in the cognitive target, symptom severity on the BDI-II, concurrent treatment, or demographics will be

For peer review only - http://bmjopen.bmj.com/site/about/guidelines.xhtml

covaried in analyses. Diagnostic status at twelve month follow-up will analysed using logistic regression. Residual depression symptoms and number of previous episodes (as an index of depression severity), and score on the AMT-AI (as an index of bias severity) at pre-intervention will be explored as moderators of treatment efficacy by computing product variables that will be analysed using linear regression. Finally, we will explore mechanisms of change by determining whether intervention effects are mediated by change on the AMT-AI and the other process measures. Indirect effects will be analyses using bootstrapped samples in PROCESS. Intent-to-treat analysis will be used for those lost to attrition by using multiple imputation to account for missing data. The number of imputed datasets will approximate the percentage of missing data (47). Any exploratory, per-protocol analyses will use data from participants who have completed six out of the eight workbook sessions. Exploratory ANOVAs will also be completed to explore between-group difference in pre-to-post intervention change on the separate AMT-AI blocks, to determine whether MemFlex may have a larger impact on specific, categoric, or alternating recall.

Trial management. The trial management committee will be formed by TD, CH, and trial statistician (PW). The trial coordinator (CH) will complete data checks to ensure data quality.

Ethics and Dissemination

This trial has been approved by the NHS National Research Ethics Committee (East of England, 11/H0305/1). All individuals will provide informed consent prior to participation, and Medical Research Council, National Health Service and professional ethical guidelines will be adhered to throughout the study. Any necessary amendments to this protocol will be published in the trial manuscript. No restrictions have been placed on the publication of results. In addition to publication of the trial paper, results will be shared in written form to trial participants, and through seminars to clinicians and the general public.

For peer review only - http://bmjopen.bmj.com/site/about/guidelines.xhtml

Safety aspects. In the unlikely occurrence of adverse events, the Medical Research Council's structured risk assessment protocols will be used to manage risk. All risk issues will be managed by the trial co-ordinator (CH), in consultation with TD, both of whom are clinical psychologists experienced in treatment of depression. Any adverse events will be reported to the research ethics committee, and will be reported in the trial manuscript.

Confidentiality and access to data. Only the trial co-ordinator and trial statistician will have access to the final dataset. Data will be securely stored in de-identified form onsite, and in encrypted electronic format. Personally identifiable information will be stored separately to the trial data, and will only be accessible via a dedicated access server. Once the data have been analysed and the trial is published, the final dataset will be deposited in de-identified form into the research unit's online data repository, along with the utilised statistical code.

Funding. This work is supported by a grant to TD from the UK Medical Research Council, grant number MC_US_A060_0019.

Trial registration. The trial protocol was registered online on 25 November 2015 at clinicaltrials.gov (identifier NCT02614326), with recruitment planned to open in January 2016. The first participant was recruited on 18 January 2016.

Discussion

Major depression is predicted to be the largest global burden of disease by 2030 (48). Reducing the recurrence of depression will therefore be a key issue for the coming years. This study will estimate the effect of a novel intervention, MemFlex, which aims to reduce negative and overgeneral memory biases, and thereby risk of depressive relapse. In simultaneously targeting these factors, the programme may reduce both direct and compounded effects on depressive relapse. This trial aims to estimate the range of likely effects of MemFlex, to provide a platform for a later stage, fully powered trial, and to begin

For peer review only

to explore mechanisms of change. If effective, MemFlex may hold promise as a low-cost and low-intensity option for reducing depressive relapse as the self-guided, workbook format of the programme would be simple and cost-effective to provide to individuals upon discharge from a treatment service, or potentially as an adjunct to complex interventions such as cognitive behavioural therapy. As the pressure that depression places on health care systems is expected to increase (49), further development of low-cost relapse prevention programmes will be vital in improving access to psychological services and quality of life for those experiencing chronic and recurrent depression.

Authors' contributions: CH co-developed the study design, co-wrote the intervention materials, and co-wrote the manuscript. SG, CO, ER, IC, KG, and JG are contributing to data collection for the trial. PW is the trial statistician. EH and AWS co-wrote the intervention materials. TD co-developed the study design, co-wrote intervention materials and co-wrote the manuscript. The authors have no conflict of interest to declare.

For peer review only

References

1. McCrone P, Dhanasiri S, Patel A, Knapp M, Lawton-Smith S. Paying the price. The cost of mental health care in England to 2026. London: King's Fund; 2008.

2. Butler AC, Chapman JE, Forman EM, Beck AT. The empirical status of cognitive-behavioral therapy: A review of meta-analyses. Clinical Psychology Review. 2006;26:17-31.

3. Judd LL. The clinical course of unipolar major depressive disorders. Archives of General Psychiatry. 1997;54(11):989-91.

4. Kessing LV. Severity of depressive episodes during the course of depressive disorder. The British Journal of Psychiatry. 2008;192:290-3.

5. Burcusa SL, Iacono WG. Risk for Recurrence in Depression. Clinical Psychology Review. 2007;27(8):959-85.

6. Conway MA, Pleydell-Pearce CW. The construction of autobiographical memories in the self-memory system. Psychological Review. 2000;107(2):261-88.

7. Dalgleish T, Werner-Seidler A. Disruptions in autobiographical memory processing in depression and the emergence of memory therapeutics. Trends in cognitive sciences. 2014;18(11):596-604.

8. Dritschel B, Beltsos S, McClintock SM. An “alternating instructions” version of the Autobiographical Memory Test for assessing autobiographical memory specificity in non-clinical populations. Memory. 2014;22:881-9.

9. Hitchcock C, Gormley S, Rees C, Rodrigues E, Gillard J, Panesar I, et al. The translational development of Memory Flexibility training (MemFlex) to enhance memory flexibility and reduce depressive symptomatology in individuals with Major Depressive Disorder. under review.

10. Dalgleish T, Bevan A, McKinnon A, Breakwell L, Mueller V, Chadwick I, et al. A comparison of MEmory Specificity Training (MEST) to education and support (ES) in the

For peer review only

treatment of recurrent depression: study protocol for a cluster randomised controlled trial. Trials. 2014;15(1):293-302.

11. Williams JMG, Barnhofer T, Crane C, Herman D, Raes F, Watkins ER, et al. Autobiographical memory specificity and emotional disorder. Psychological Bulletin. 2007;133:122-48.

12. Goddard L, Dritschel B, Burton A. Role of autobiographical memory in social problem solving and depression. J Abnorm Psychol. 1996;105.

13. Klein SB, Cosmides L, Tooby J, Chance S. Decisions and the evolution of memory: multiple systems, multiple functions. Psychological review. 2002;109(2):306.

14. Klein SB, Cosmides L, Tooby J, Chance S. Priming exceptions: A test of the scope hypothesis in naturalistic trait judgments. Social Cognition. 2001;19(4):443-68.

15. Hipwell AE, Sapotichne B, Klostermann S, Battista D, Keenan K. Autobiographical memory as a predictor of depression vulnerability in girls. Journal of Clinical Child & Adolescent Psychology. 2011;40:254-65.

16. Sumner JA, Griffith JW, Mineka S. Overgeneral autobiographical memory as a predictor of the course of depression: A meta-analysis. Behaviour Research and Therapy. 2010;48(7):614-25.

17. Gotlib I, Joormann J. Cognition and depression: current status and future directions. Annu Rev Clin Psychol. 2010;6:285.

18. Werner-Seidler A, Moulds ML. Autobiographical memory characteristics in depression vulnerability: Formerly depressed individuals recall less vivid positive memories. Cognition & emotion. 2011;25(6):1087-103.

19. Joormann J, Siemer M. Memory accessibility, mood regulation, and dysphoria: difficulties in repairing sad mood with happy memories? J Abnorm Psychol. 2004;113(2):179.

For peer review only

20. Teasdale JD, Dent J. Cognitive vulnerability to depression: An investigation of two hypotheses. British Journal of Clinical Psychology. 1987;26:113-26.

21. Beck AT. Depression: Clinical, Experimental, and Theoretical Aspects: Hoeber Medical Division, Harper & Row; 1967.

22. Williams JMG, Teasdale JD, Segal Z, Soulsby J. Mindfulness-based cognitive therapy reduces overgeneral autobiographical memory in formerly depressed patients. J Abnorm Psychol. 2000;109.

23. Watkins ER, Taylor RS, Byng R, Baeyens C, Read R, Pearson K, et al. Guided self-help concreteness training as an intervention for major depression in primary care: a Phase II randomized controlled trial. Psychological Medicine. 2012;42(07):1359-71.

24. Nolen-Hoeksema S. Responses to depression and their effects on the duration of depressive episodes. Journal of Abnormal Psychology. 1991;100(4):569-82.

25. Beike DR, Brandon NR, Cole HE. Is Sharing Specific Autobiographical Memories a Distinct Form of Self-Disclosure? Journal of Experimental Psychology: General. 2016;145:434-50.

26. Jing HG, Madore KP, Schacter DL. Worrying About the Future: An Episodic Specificity Induction Impacts Problem Solving, Reappraisal, and Well-Being. Journal of Experimental Psychology: General. 2016;145:402-18.

27. Hitchcock C, Rees C, Dalgleish T. The Devil's in the Detail: Accessibility of Specific Personal Memories Supports Rose-Tinted Self-Generalizations in Mental Health and Toxic Self-Generalizations in Clinical Depression. Journal of Experimental Psychology: General. 2017:No Pagination Specified.

28. Dritschel B, Beltsos S, McClintock SM. An "alternating instructions" version of the Autobiographical Memory Test for assessing autobiographical memory specificity in non-clinical populations. Memory. 2013;22(8):881-9.

For peer review only

29. Sumner JA, Griffith JW, Mineka S, Rekart KN, Zinbarg RE, Craske MG. Overgeneral autobiographical memory and chronic interpersonal stress as predictors of the course of depression in adolescents. Cognition and Emotion. 2011;25(1):183-92.

30. Neshat-Doost HT, Dalgleish T, Yule W, Kalantari M, Ahmadi SJ, Dyregrov A, et al. Enhancing Autobiographical Memory Specificity Through Cognitive Training: An Intervention for Depression Translated From Basic Science. Clinical Psychological Science. 2013;1(1):84-92.

31. Raes F, Williams JMG, Hermans D. Reducing cognitive vulnerability to depression: A preliminary investigation of MEmory Specificity Training (MEST) in inpatients with depressive symptomatology. Journal of behavior therapy and experimental psychiatry. 2009;40(1):24-38.

32. Hitchcock C, Werner-Seidler A, Blackwell SE, Dalgleish T. Autobiographical episodic memory-based training for the treatment of mood, anxiety and stress-related disorders: A systematic review and meta-analysis. Clinical Psychology Review. 2017;52:92-107.

33. Nolen-Hoeksema S, Wisco BE, Lyubomirsky S. Rethinking Rumination. Perspectives on Psychological Science. 2008;3(5):400-24.

34. Taylor SE, Brown JD. Illusion and well-being: A social psychological perspective on mental health. Psychological Bulletin. 1988;103(2):193-210.

35. Hitchcock C, Mueller V, Hammond E, Rees C, Werner-Seidler A, Dalgleish T. The effects of autobiographical memory flexibility (MemFlex) training: An uncontrolled trial in individuals in remission from depression. Journal of Behavior Therapy and Experimental Psychiatry. 2016;52:92-8.

36. Hitchcock C, Hammond E, Rees C, Panesar I, Watson P, Werner-Seidler A, et al. Memory Flexibility training (MemFlex) to reduce depressive symptomatology in individuals

For peer review only

with major depressive disorder: study protocol for a randomised controlled trial. Trials. 2015;16(1):1-8.

37. Medical Research Council. A Framework for Development and Evaluation of RCTs for Complex Interventions to Improve Health. London: Medical Research Council; 2000.

38. First M, Williams JBW, Karg RS, Spitzer R. Structured Clinical Interview for DSM-V Disorders (SCID-V). Arlington, VA: American Psychiatric Association; 2015.

39. Keller MB, Lavori PW, Friedman B, et al. The longitudinal interval follow-up evaluation: A comprehensive method for assessing outcome in prospective longitudinal studies. Archives of General Psychiatry. 1987;44(6):540-8.

40. Devilly GJ, Borkovec TD. Psychometric properties of the credibility/expectancy questionnaire. Journal of Behavior Therapy and Experimental Psychiatry. 2000;31(2):73-86.

41. Beck AT, Steer R, Brown G. Manual for the Beck Depression Inventory-II. San Antonio, TX:: Psychological Corporation; 1996.

42. Treynor W, Gonzalez R, Nolen-Hoeksema S. Rumination reconsidered: A psychometric analysis. Cogn Ther Res. 2003;27(3):247-59.

43. Sexton KA, Dugas MJ. The cognitive avoidance questionnaire: validation of the English translation. Journal of Anxiety Disorders. 2008;22(3):355-70.

44. Lyubomirsky S, Nolen-Hoeksema S. Effects of self-focused rumination on negative thinking and interpersonal problem solving. Journal of Personality and Social Psychology. 1995;69(1):176-90.

45. Tombaugh TN, Kozak J, Rees L. Normative Data Stratified by Age and Education for Two Measures of Verbal Fluency: FAS and Animal Naming. Archives of Clinical Neuropsychology. 1999;14(2):167-77.

For peer review only

46. Travers-Hill E, Dunn BD, Hoppitt L, Hitchcock C, Dalgleish T. Beneficial effects of training in self-distancing and perspective broadening for people with a history of recurrent depression. Behaviour Research and Therapy. 2017;95:19-28.

47. Bodner TE. What Improves with Increased Missing Data Imputations? Structural Equation Modeling: A Multidisciplinary Journal. 2008;15(4):651-75.

48. Mathers C, Fat DM, Boerma JT. The global burden of disease: 2004 update: World Health Organization; 2008.

49. Promoting Mental Health; Concepts emerging evidence and practice. Summary report. Geneva: World Health Organisation; 2004.

For peer review only

Figure Legends.

Figure 1. Flow chart of study design (CONSORT diagram). All outcome and process measures will be completed at all time points.

For peer review only

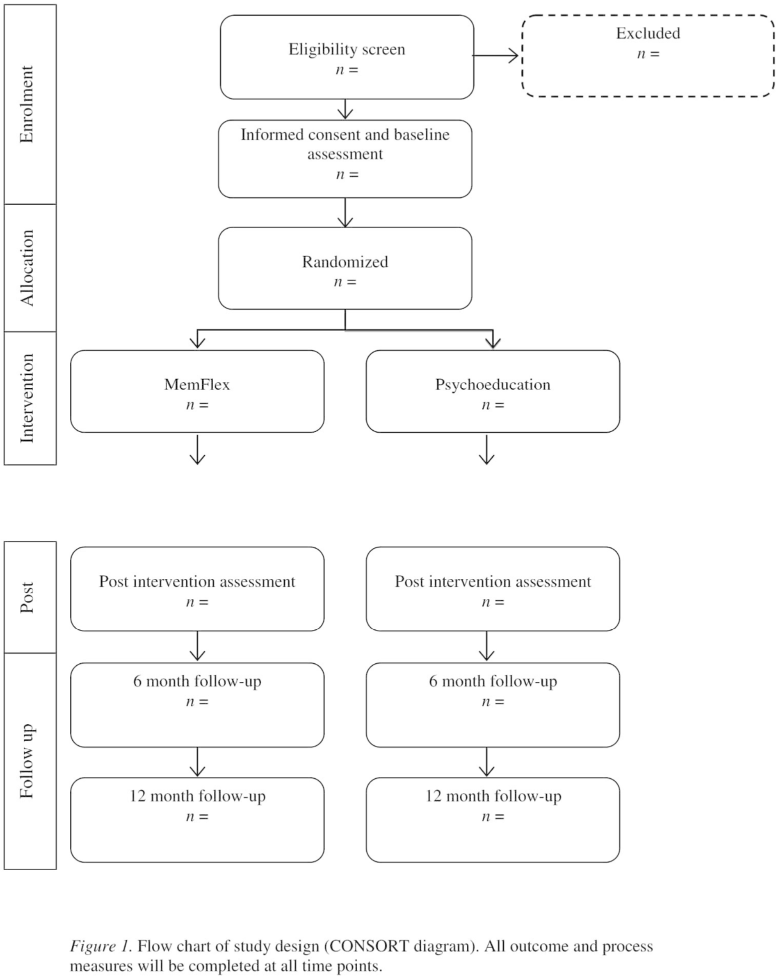

Figure 1. Flow chart of study design (CONSORT diagram). All outcome and process measures will be completed at all time points.

140x198mm (300 x 300 DPI)

SPIRIT

STANDARD PROTOCOL ITEMS: RECOMMENDATIONS FOR INTERVENTIONAL TRIALS

SPIRIT 2013 Checklist: Recommended items to address in a clinical trial protocol and related documents*

Section/item	Item No	Description	Addressed on page number
**Administrative information**			
Title	1	Descriptive title identifying the study design, population, interventions, and, if applicable, trial acronym	_______1________
Trial registration	2a	Trial identifier and registry name. If not yet registered, name of intended registry	_______2_______
	2b	All items from the World Health Organization Trial Registration Data Set	_2 (refer to online record)________
Protocol version	3	Date and version identifier	_______1________
Funding	4	Sources and types of financial, material, and other support	______15_______
Roles and responsibilities	5a	Names, affiliations, and roles of protocol contributors	___1, 16_______
	5b	Name and contact information for the trial sponsor	____1,15________
	5c	Role of study sponsor and funders, if any, in study design; collection, management, analysis, and interpretation of data; writing of the report; and the decision to submit the report for publication, including whether they will have ultimate authority over any of these activities	____16________
	5d	Composition, roles, and responsibilities of the coordinating centre, steering committee, endpoint adjudication committee, data management team, and other individuals or groups overseeing the trial, if applicable (see Item 21a for data monitoring committee)	______14_______

For peer review only - http://bmjopen.bmj.com/site/about/guidelines.xhtml

Introduction

Background and rationale	6a	Description of research question and justification for undertaking the trial, including summary of relevant studies (published and unpublished) examining benefits and harms for each intervention	_____4-7___
	6b	Explanation for choice of comparators	____7______
Objectives	7	Specific objectives or hypotheses	____7________
Trial design	8	Description of trial design including type of trial (eg, parallel group, crossover, factorial, single group), allocation ratio, and framework (eg, superiority, equivalence, noninferiority, exploratory)	___8_______

Methods: Participants, interventions, and outcomes

Study setting	9	Description of study settings (eg, community clinic, academic hospital) and list of countries where data will be collected. Reference to where list of study sites can be obtained	___8________
Eligibility criteria	10	Inclusion and exclusion criteria for participants. If applicable, eligibility criteria for study centres and individuals who will perform the interventions (eg, surgeons, psychotherapists)	____8_______
Interventions	11a	Interventions for each group with sufficient detail to allow replication, including how and when they will be administered	__9-11_______
	11b	Criteria for discontinuing or modifying allocated interventions for a given trial participant (eg, drug dose change in response to harms, participant request, or improving/worsening disease)	_____NA______
	11c	Strategies to improve adherence to intervention protocols, and any procedures for monitoring adherence (eg, drug tablet return, laboratory tests)	_____10______
	11d	Relevant concomitant care and interventions that are permitted or prohibited during the trial	______8______
Outcomes	12	Primary, secondary, and other outcomes, including the specific measurement variable (eg, systolic blood pressure), analysis metric (eg, change from baseline, final value, time to event), method of aggregation (eg, median, proportion), and time point for each outcome. Explanation of the clinical relevance of chosen efficacy and harm outcomes is strongly recommended	_____11-12____
Participant timeline	13	Time schedule of enrolment, interventions (including any run-ins and washouts), assessments, and visits for participants. A schematic diagram is highly recommended (see Figure)	_____Figure 1__

For peer review only - http://bmjopen.bmj.com/site/about/guidelines.xhtml

Sample size	14	Estimated number of participants needed to achieve study objectives and how it was determined, including clinical and statistical assumptions supporting any sample size calculations	_____13_____
Recruitment	15	Strategies for achieving adequate participant enrolment to reach target sample size	____8_______
**Methods: Assignment of interventions (for controlled trials)**			
Allocation:			
Sequence generation	16a	Method of generating the allocation sequence (eg, computer-generated random numbers), and list of any factors for stratification. To reduce predictability of a random sequence, details of any planned restriction (eg, blocking) should be provided in a separate document that is unavailable to those who enrol participants or assign interventions	______9______
Allocation concealment mechanism	16b	Mechanism of implementing the allocation sequence (eg, central telephone; sequentially numbered, opaque, sealed envelopes), describing any steps to conceal the sequence until interventions are assigned	_____9____
Implementation	16c	Who will generate the allocation sequence, who will enrol participants, and who will assign participants to interventions	___9______
Blinding (masking)	17a	Who will be blinded after assignment to interventions (eg, trial participants, care providers, outcome assessors, data analysts), and how	___9, 13________
	17b	If blinded, circumstances under which unblinding is permissible, and procedure for revealing a participant's allocated intervention during the trial	_____NA_______
**Methods: Data collection, management, and analysis**			
Data collection methods	18a	Plans for assessment and collection of outcome, baseline, and other trial data, including any related processes to promote data quality (eg, duplicate measurements, training of assessors) and a description of study instruments (eg, questionnaires, laboratory tests) along with their reliability and validity, if known. Reference to where data collection forms can be found, if not in the protocol	__11-12___
	18b	Plans to promote participant retention and complete follow-up, including list of any outcome data to be collected for participants who discontinue or deviate from intervention protocols	____11-12____

For peer review only - http://bmjopen.bmj.com/site/about/guidelines.xhtml

Data management	19	Plans for data entry, coding, security, and storage, including any related processes to promote data quality (eg, double data entry; range checks for data values). Reference to where details of data management procedures can be found, if not in the protocol	___13____
Statistical methods	20a	Statistical methods for analysing primary and secondary outcomes. Reference to where other details of the statistical analysis plan can be found, if not in the protocol	___13-14_______
	20b	Methods for any additional analyses (eg, subgroup and adjusted analyses)	__13-14________
	20c	Definition of analysis population relating to protocol non-adherence (eg, as randomised analysis), and any statistical methods to handle missing data (eg, multiple imputation)	_____14______
**Methods: Monitoring**			
Data monitoring	21a	Composition of data monitoring committee (DMC); summary of its role and reporting structure; statement of whether it is independent from the sponsor and competing interests; and reference to where further details about its charter can be found, if not in the protocol. Alternatively, an explanation of why a DMC is not needed	__14_______
	21b	Description of any interim analyses and stopping guidelines, including who will have access to these interim results and make the final decision to terminate the trial	_____14______
Harms	22	Plans for collecting, assessing, reporting, and managing solicited and spontaneously reported adverse events and other unintended effects of trial interventions or trial conduct	_____15_____
Auditing	23	Frequency and procedures for auditing trial conduct, if any, and whether the process will be independent from investigators and the sponsor	______11_____
**Ethics and dissemination**			
Research ethics approval	24	Plans for seeking research ethics committee/institutional review board (REC/IRB) approval	_____14______
Protocol amendments	25	Plans for communicating important protocol modifications (eg, changes to eligibility criteria, outcomes, analyses) to relevant parties (eg, investigators, REC/IRBs, trial participants, trial registries, journals, regulators)	______14______

For peer review only - http://bmjopen.bmj.com/site/about/guidelines.xhtml

Consent or assent	26a	Who will obtain informed consent or assent from potential trial participants or authorised surrogates, and how (see Item 32)	_____9______
	26b	Additional consent provisions for collection and use of participant data and biological specimens in ancillary studies, if applicable	____NA______
Confidentiality	27	How personal information about potential and enrolled participants will be collected, shared, and maintained in order to protect confidentiality before, during, and after the trial	____15______
Declaration of interests	28	Financial and other competing interests for principal investigators for the overall trial and each study site	____16_______
Access to data	29	Statement of who will have access to the final trial dataset, and disclosure of contractual agreements that limit such access for investigators	_____15_____
Ancillary and post-trial care	30	Provisions, if any, for ancillary and post-trial care, and for compensation to those who suffer harm from trial participation	____15______
Dissemination policy	31a	Plans for investigators and sponsor to communicate trial results to participants, healthcare professionals, the public, and other relevant groups (eg, via publication, reporting in results databases, or other data sharing arrangements), including any publication restrictions	____15______
	31b	Authorship eligibility guidelines and any intended use of professional writers	_______16_____
	31c	Plans, if any, for granting public access to the full protocol, participant-level dataset, and statistical code	_____NA______
**Appendices**			
Informed consent materials	32	Model consent form and other related documentation given to participants and authorised surrogates	_Available on request_
Biological specimens	33	Plans for collection, laboratory evaluation, and storage of biological specimens for genetic or molecular analysis in the current trial and for future use in ancillary studies, if applicable	_____NA______

*It is strongly recommended that this checklist be read in conjunction with the SPIRIT 2013 Explanation & Elaboration for important clarification on the items. Amendments to the protocol should be tracked and dated. The SPIRIT checklist is copyrighted by the SPIRIT Group under the Creative Commons "Attribution-NonCommercial-NoDerivs 3.0 Unported" license.

For peer review only - http://bmjopen.bmj.com/site/about/guidelines.xhtml
